# Facilitating rapid access to addiction treatment: a randomized controlled trial

**DOI:** 10.1186/s13722-021-00240-y

**Published:** 2021-05-25

**Authors:** Anita Srivastava, Sarah Clarke, Kate Hardy, Meldon Kahan

**Affiliations:** 1 Unity Health, Family Medicine, 1st Floor, 30 The Queensway, Toronto, ON M6R 1B5 Canada; 2 META:PHI, Women’s College Hospital, Toronto, ON M5S 1B2 Canada; 3 Substance Use Service, Women’s College Hospital, 3rd Floor, Toronto, ON M5S 1B2 Canada

**Keywords:** Treatment access, Alcohol use disorder, Opioid use disorder, ED visits

## Abstract

**Background:**

Obtaining timely access to addiction medicine treatment for patients with substance use disorders is challenging and patients often have to navigate complex referral pathways. This randomized controlled trial examines the effect of providing an expedited pathway to addiction medicine treatment on initial treatment engagement and health care utilization.

**Methods:**

Individuals with possible alcohol or opioid use disorder were recruited from three residential withdrawal management services (WMS). Subjects randomized to the Delayed Intervention (DI) group were given contact information for a nearby addiction medicine clinic; those randomized to the Rapid Intervention (RI) group were given an appointment at the clinic within 2 days and were accompanied to their first appointment.

**Results:**

Of the 174 individuals who were screened, 106 were randomized to either the DI or RI group. The two groups were similar in demographics, housing status, and substance use in the last 30 days. In the 6-month period following randomization, 85% of the RI group attended at least one clinic appointment, compared to only 29% in the DI group (p < 0.0001). The RI group had a mean of 6.39 ED visits per subject in the 12 months after randomization, while the DI group had a mean of 13.02 ED visits per subject in the same 12-month period (p = 0.0469). Other health utilization measures did not differ between the two groups.

**Conclusion:**

Providing immediate facilitated access to an addiction medicine service resulted in greater initial engagement and reduced emergency department visits at 6 months.

*Trial registration* This trial is registered at the National Institutes of Health (ClinicalTrials.gov) under identifier #NCT01934751.

**Supplementary Information:**

The online version contains supplementary material available at 10.1186/s13722-021-00240-y.

## Background

Substance use disorders are a major cause of morbidity, mortality, and health care utilization in Canada. The Canadian Centre on Substance Use and Addiction found the total cost of substance use in Canada to be $38.4 billion in 2014 [1]. Alcohol was the leading contributor to these costs (38.1%), while opioids were third (9.1%) behind tobacco (31.2%). In spite of the significant burden of illness from alcohol use disorder (AUD) and opioid use disorder (OUD), access to treatment is often severely limited by lengthy wait lists and complex intake procedures.

Additionally, although there are effective pharmacological interventions for AUD and OUD, patients face barriers to accessing these medications. Oral naltrexone^1^ and acamprosate are effective first-line treatments for AUD [2, 3], and opioid agonist therapy (OAT) with buprenorphine or methadone is very effective for OUD [4]; however, these medications are not routinely prescribed [5–7]. Controlled trials have shown that people who use heroin who receive buprenorphine in the emergency department (ED) are far more likely to be retained in OAT than as if they are simply referred to an outpatient addiction clinic [8, 9]. However, substance use treatment is rarely initiated in the ED, where patients with substance use disorders are frequent visitors [10], even if the patient presents with a life-threatening complication of their substance use. EDs sometimes refer patients with substance use disorders to non-medical, community-based residential withdrawal management services (WMS), especially if they have unstable housing. However, WMS have high rates of readmission because they are often unable to transition patients to formal treatment programs [11, 12]. Furthermore, staff at non-medical WMS (and at many abstinence-based inpatient addiction treatment programs) cannot prescribe medications to clients.

Treatment retention rates are inversely correlated with length of time between assessment and treatment initiation [13]. In 2018, the average wait time for residential addiction treatment programs was 50 days, an increase from 43 days in 2015 [14]. Furthermore, many people who use substances find it difficult to make and keep appointments because they lack social support, do not have telephones, lack funds for transportation, or have unstable housing [15].

Program wait times and requirements reduce the likelihood that patients will engage in treatment [13, 16, 17]. People with AUD and OUD often experience a cycle of using, facing a crisis, seeking help, being unable to access treatment, and relapsing, leading to frequent ED use [10, 18]. Between 2015 and 2018, ED visits for substance use disorders increased by 40% in Ontario, compared to a 6% overall increase in ED visits; and repeat ED visits within 30 days for a substance use disorder increased by 50% [14].

This trial tested the hypothesis that individuals with possible AUD or OUD residing in a non-medical WMS given rapid and facilitated access to medical treatment at an addiction clinic would have greater treatment engagement and better health care outcomes than individuals with usual access to treatment.

## Methods

### Study design

In this randomized controlled parallel-group trial, potential participants were identified by staff at three non-medical residential WMS sites in downtown Toronto. The WMS staff alerted the study team when a potential participant was identified; the research assistant was available from Monday to Friday during business hours and would travel to the WMS to ensure that the potential participant satisfied the inclusion criteria and to obtain their consent. Having possible AUD, determined by an Alcohol Use Disorders Identification Test (AUDIT) score of at least eight [19], or possible OUD, determined by self-identified recent use and at least one harmful consequence of use, was a requisite criterion.^2^ Anyone who was actively in opioid agonist treatment was excluded (see Additional file 1: Appendix S1). Eligible participants were consented by the RA and randomized using the Medidata Rave data collection tool (https://ecog-acrin.org/resources/medidata-rave) to the Delayed Intervention (DI) group or the Rapid Intervention (RI) group, with an allocation ratio of one to one.

Participants in the RI group had facilitated access to an appointment with an addiction physician at one of two addiction medicine clinics located within a few kilometers of the WMS site arranged for them by the RA. Facilitated access involved receiving an appointment within 1–3 days of enrollment and accompaniment to their initial appointment by the RA. They were also given local transit tokens for future appointments. Participants in the DI group were offered an appointment with an addiction physician but were given a card with the phone number of the addiction medicine clinic and asked to call and arrange their own appointment.

At the addiction medicine clinics, all study participants were assessed by an addiction physician, who offered pharmacotherapy where appropriate, solution-focused counselling, and a referral to a primary care physician if needed. Ongoing care with the addiction medicine clinic was available to all participants.

The primary outcome measure was the proportion of participants in each group attending at least one appointment at the addiction medicine clinic in the 6 months after initial randomization.

The secondary outcome measures were changes in health care utilization. Provincial databases managed by the Institute for Clinical and Evaluative Sciences (ICES) were used to measure changes in health care utilization from one year pre-intervention to one year post-intervention*.* Health care utilization included ED visits, hospital admissions, length of hospital stays, number of primary care visits, laboratory usage, and prescriptions for naltrexone or acamprosate for AUD and methadone or buprenorphine for OUD.

The study, which took place between January and December 2014, was conducted in accordance with the Tri-Council Policy Statement: Ethical Conduct for Research Involving Humans (TCPS 2) and was approved by the Research Ethics Boards of St. Michael’s Hospital, University Health Network, and Women’s College Hospital.

### Statistical analysis

The sample size estimation and statistical analysis were performed by the Applied Health Research Centre (AHRC) and the Institute for Clinical Evaluative Studies (ICES), and interpreted with the assistance of Chris Meaney, Department of Family and Community Medicine, University of Toronto. Based on our clinical experience, we estimated that 10% of participants in the DI group would attend an appointment with the addiction service, compared with 30% of participants in the RI group and by setting our power at 0.8 calculated a total sample size of 124.

The primary analysis was intention-to-treat and involved all participants. The analysis was performed by AHRC using a Pearson’s Chi-squared test with Yates’ continuity correction.

The secondary analyses were also intention-to-treat and involved participants who consented to provide their Ontario Health Insurance Plan (OHIP) number. A regression analysis using a quasi-Poisson model was performed on health care utilization using count-based outcome visits from the following ICES databases: Discharge Abstract Database (DAD), Ontario Drug Benefits (ODB), and National Ambulatory Care Reporting Service (NACRS).

## Results

**Fig. 1:**
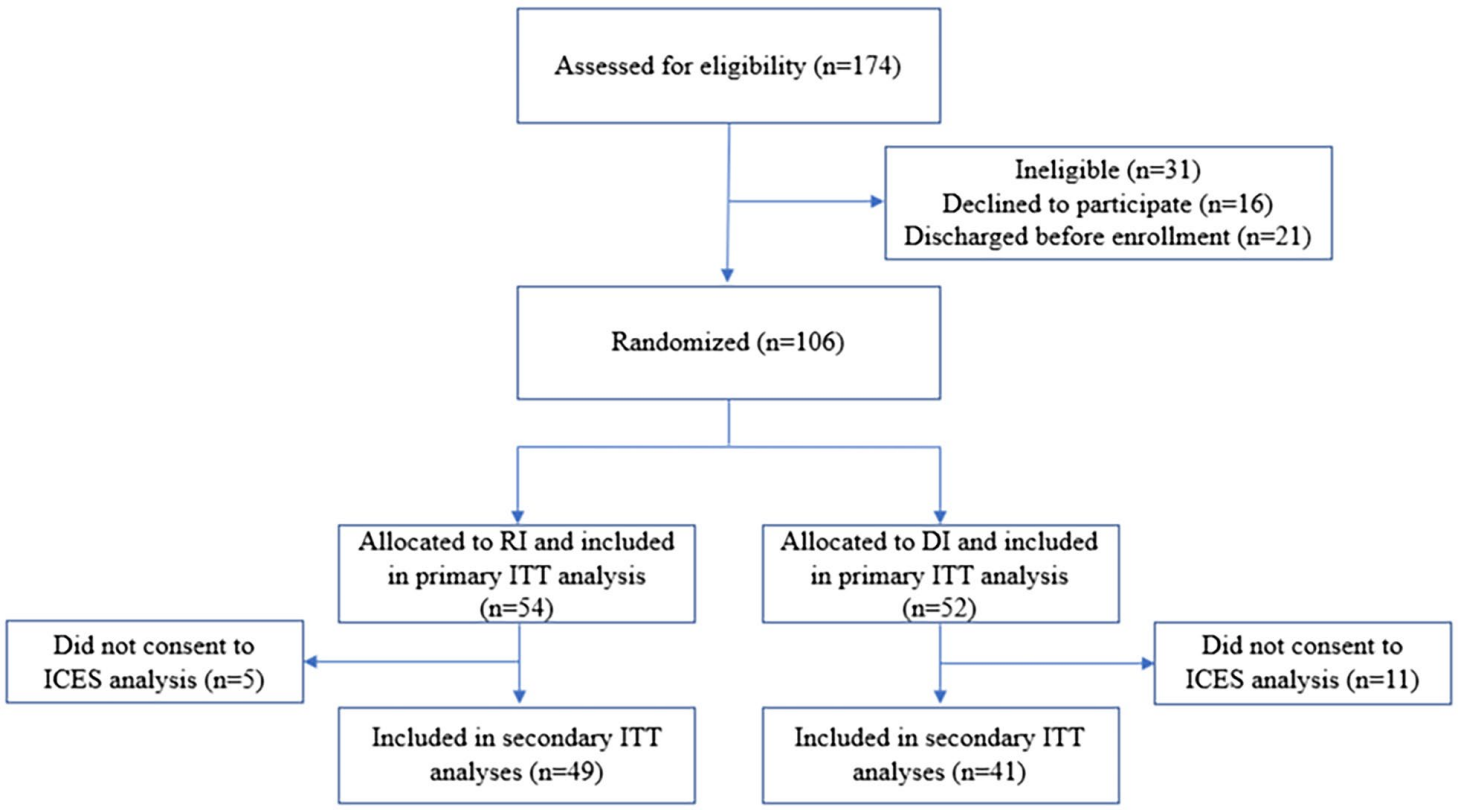
Allocation

A total of 174 individuals at the three WMS sites were screened for eligibility. Of those screened, 106 met eligibility criteria and were randomized into the RI or DI group. Fifty-four participants were randomized to the RI group and 52 were randomized to the DI group, all of whom were included in the primary analysis; within these cohorts, 49 members of the RI group (91%) and 41 members of the DI group (79%) consented to inclusion in the secondary analyses (see Fig. 1). Delays in obtaining Research Ethics Board approvals shortened the study timeline and did not allow us to meet our target enrollment of 124.

The baseline demographics of the two groups were similar in terms of sex, age, marital status, housing within the past month, and primary dependency, as shown in Table 1.

**Table 1:** Baseline demographics

Characteristics	DI (N = 52)	RI (N = 54)	P-value
Female	48% (25)	44% (24)	0.707668
Male	52% (27)	56% (30)	
Age	40.25 ± 10.10	41.16 ± 11.26	
Single	79% (41)	83% (45)	0.555001
Married/cohabitating	21% (11)	17% (9)	
Home	58% (30)	56% (30)	0.562197
With family or friends	15% (8)	24% (13)	
Hospital or treatment centre	12% (6)	4% (2)	
Street or shelter	12% (6)	9% (5)	
Jail	2% (1)	4% (2)	
Multiple places	2% (1)	4% (2)	
Primary dependency alcohol	77% (40)	74% (40)	0.733246
Primary dependency opioids	23% (12)	26% (14)	

The proportion of participants in each group attending at least one appointment at the addiction medicine clinic within 6 months of randomization was compared between the two arms (Table 2): 85% of the RI group had at least one appointment at the addiction medicine clinic in the 6 months post-randomization compared to only 29% in the DI group (p-value < 0.0001).

**Table 2:** Attendance at addiction medicine clinic

Clinic attendance	DI (N = 52)	RI (N = 54)	p-value (with Yates’ continuity correction)
Yes	29% (15)	85% (46)	< 0.0001
No	71% (37)	15% (8)	

Although the rates of attendance were different between the two arms, the rates of retention were not: nine of the fifteen (60%) DI group attendees and 27 of the 46 (59%) RI group attendees attended more than one appointment.

Analysis of a provincial database (DAD) showed that ED visits at 12 months decreased in the RI group compared to the DI group. Participants in the two groups had a similar mean of ED visits in the 12 months pre-randomization (9.43 for the DI group and 9.12 for the RI group); however, the RI group had a mean of 6.39 ED visits in the twelve months after randomization, while the DI group had a mean of 13.02 ED visits in the same 12-month period (Table 3).

**Table 3:** Differences in ED visits pre- and post-randomization

	DI (N = 41)	RI (N = 49)
12 months pre-randomization	387 (9.43)	447 (9.12)
12 months post-randomization	534 (13.02)	313 (6.39)

A regression analysis using a quasi-Poisson model was performed on this data (Table 4), and the decrease in ED visits in the RI versus the DI group was found to be significant (p = 0.0469).

**Table 4:** Quasi-Poisson with Pearson scale residuals using DAD data, N = 90

	Estimate regression coefficient	Wald 95% confidence limits		p-value
Intercept	1.1749	0.7191	1.7306	< 0.0001
DI	0.5211	0.0072	1.0351	0.0469
RI	0.0000	0.0000	0.0000	
Pre-randomization ED visits	0.0367	0.0300	0.0434	< 0.0001

Pre- and post-randomization rates of other health utilization measures, including hospitalizations, length of hospitalizations, primary care visits, and lab utilization, did not differ significantly between the two groups, as shown in Table 5 (the total for the cohort is followed by the mean per participant in parentheses).

**Table 5:** Differences in health care utilization pre- and post-randomization

	DI (N = 41)	RI (N = 49)
Hospitalizations		
12 months pre-randomization	30 (0.75)	40 (0.82)
12 months post-randomization	35 (0.85)	27 (0.55)
Days in hospital		
12 months pre-randomization	225 (5.49)	209 (4.27)
12 months post-randomization	290 (7.07)	228 (4.65)
Primary care visits		
12 months pre-randomization	421 (10.27)	487 (9.94)
12 months post-randomization	778 (18.98)	653 (13.33)
Outpatient laboratory services		
12 months pre-randomization	662 (16.15)	595 (12.14)
12 months post-randomization	846 (20.63)	896 (18.29)

## Discussion

The RI group was more likely than the DI group to attend at least one appointment at the addiction medicine clinic. Only 60% of the participants in each group who attended the initial appointment attended a second appointment, suggesting that ongoing facilitated appointments could be beneficial. However, it could also mean that participants were no longer in the geographic vicinity of the clinic and had sought care at other addiction services after the initial clinic visit.

The addiction medicine clinics involved in this trial usually prescribe medications for AUD and OUD on the first visit. This could have contributed to the reduction in ED visits in the RI group; use of medications for AUD and OUD are associated with significant declines in ED visits and hospitalizations [20–24]. However, this finding, while promising, requires confirmation with a larger trial. Results might have been skewed if a few very heavy ED users had been allocated to the DI group by chance. Also, other secondary outcomes did not differ significantly between the two groups, making it unclear why ED visits would differ between the two groups.

One limitation of this trial is its small size. An additional limitation is the absence of follow-up data from participants, which would provide some context to the secondary outcomes as well as the factors involved in treatment engagement. Further research that addresses these two issues is needed.

This trial supports research showing that rapid, facilitated access to addiction medicine enhances treatment retention and treatment effectiveness. The preliminary results of this trial led to the creation of the Mentoring, Education, and Clinical Tools for Addiction: Primary Care–Hospital Integration (META:PHI) project, a provincial initiative to spread this rapid access model of addiction care. Some of the clinics created following this initiative have demonstrated positive patient outcomes [25–27]. There are several ways that on-site and immediate access can be accomplished in addition to rapid access clinics. WMS and psychosocial treatment programs should have physicians and nurse practitioners on staff who can prescribe medications for AUD and OUD. EDs, inpatient hospital units, and primary care clinics should be able to initiate these treatments without having to refer patients to a specialized, off-site clinic.

## Conclusion

Rapid, facilitated access to addiction medicine treatment increases initial treatment engagement and reduces ED visits in patients with possible AUD or OUD who are residing in a non-medical, community-based withdrawal management center. Larger trials are needed to confirm these findings. The health care system should ensure immediate, on-site access to medication for patients with substance use disorders.

## Supplementary Information

**Additional file 1: Appendix S1. **Eligibility screening questionnaires.

## Data Availability

The following datasets are available from the Canadian Institute for Health Information: DAD: https://www.cihi.ca/en/discharge-abstract-database-metadata-dad. NACRS: https://www.cihi.ca/en/national-ambulatory-care-reporting-system-metadata-nacrs. The ODB dataset is maintained by the Ontario Data Catalogue and is not publicly available: https://data.ontario.ca/dataset/ontario-drug-benefit-odb-database.

## Abbreviations

AHRC: Applied Health Research Centre
AUD: Alcohol use disorder
DAD: Discharge abstract database
DI: Delayed intervention
ED: Emergency department
ICES: Institute for Clinical and Evaluative Sciences
NACRS: National Ambulatory Care Reporting Service
OAT: Opioid agonist therapy
ODB: Ontario drug benefits
OUD: Opioid use disorder
RI: Rapid intervention
WMS: Withdrawal management services

## Footnotes

1 Extended-release intramuscular naltrexone is currently not available in Canada.

2 The non-clinical research assistant was not qualified to diagnose participants with AUD or OUD. The investigators determined that these screening tools were adequate to identify possible use disorders. The diagnosis was confirmed (without any disparities found) by medical staff using standard DSM criteria upon admission to the medical hospital-based program.

## References

[1] Canadian Substance Use Costs and Harms Scientific Working Group. Canadian substance use costs and harms (2007–2014). Ottawa, ON: Canadian Centre on Substance Use and Addiction. 2018. https://www.ccsa.ca/sites/default/files/2019-04/CSUCH-Canadian-Substance-Use-Costs-Harms-Report-2018-en.pdf.

[2] Snyder JL, Bowers T (2008). The efficacy of acamprosate and naltrexone in the treatment of alcohol dependence: a relative benefits analysis of randomized controlled trials. Am J Drug Alcohol Abuse.

[3] Rosner S, Leucht S, Lehert P, Soyka M (2008). Acamprosate supports abstinence, naltrexone prevents excessive drinking: evidence from a meta-analysis with unreported outcomes. J Psychopharmacol.

[4] Mattick RP, Breen C, Kimber J, Davoli M (2014). Buprenorphine maintenance versus placebo or methadone maintenance for opioid dependence. Cochrane Database Syst Rev.

[5] Spithoff S, Turner S, Gomes T, Martins D, Singh S (2017). First-line medications for alcohol use disorders among public drug plan beneficiaries in Ontario. Can Fam Physician.

[6] Huhn AS, Dunn KE (2017). Why aren’t physicians prescribing more buprenorphine?. J Subst Abuse Treat.

[7] Volkow ND, Frieden TR, Hyde PS, Cha SS (2014). Medication-assisted therapies—tackling the opioid-overdose epidemic. N Engl J Med.

[8] D'Onofrio G, O'Connor PG, Pantalon MV, Chawarski MC, Busch SH, Owens PH (2015). Emergency department-initiated buprenorphine/naloxone treatment for opioid dependence: a randomized clinical trial. JAMA.

[9] Srivastava A, Kahan M, Njoroge I, Sommer LZ (2019). Buprenorphine in the emergency department: Randomized clinical controlled trial of clonidine versus buprenorphine for the treatment of opioid withdrawal. Can Fam Physician.

[10] Kim JJ, Kwok ESH, Cook OG, Calder LA (2018). Characterizing highly frequent users of a large canadian urban emergency department. West J Emerg Med.

[11] Haley SJ, Dugosh KL, Lynch KG (2011). Performance contracting to engage detoxification-only patients into continued rehabilitation. J Subst Abuse Treat.

[12] McLellan AT, Weinstein RL, Shen Q, Kendig C, Levine M (2005). Improving continuity of care in a public addiction treatment system with clinical case management. Am J Addict.

[13] Hoffman KA, Ford JH, Tillotson CJ, Choi D, McCarty D (2011). Days to treatment and early retention among patients in treatment for alcohol and drug disorders. Addict Behav.

[14] Office of the Auditor General of Ontario Place of publication: Toronto, ON. Annual Report, vol. 1, sect. 3.02. Queen’s Printer for Ontario; 2019. https://www.auditor.on.ca/en/content/annualreports/arreports/en19/v1_302en19.pdf.

[15] Palepu A, Gadermann A, Hubley A, Farrell S, Gogosis E, Aubry T (2013). Substance use and access to health care and addiction treatment among homeless and vulnerably housed persons in three Canadian cities. PLoS ONE.

[16] Redko C, Rapp RC, Carlson RG (2006). Waiting time as a barrier to treatment entry: perceptions of substance users. J Drug Issues.

[17] Pollini RA, McCall L, Mehta SH, Vlahov D, Strathdee SA (2006). Non-fatal overdose and subsequent drug treatment among injection drug users. Drug Alcohol Depend.

[18] Canadian Institute for Health Information. Common Challenges, Shared Priorities: Measuring Access to Home and Community Care and to Mental Health and Addictions Services in Canada, Nov 2019, Ottawa, ON. 2019. https://www.cihi.ca/sites/default/files/document/shp-companion-report-en.pdf.

[19] Moehring A, Rumpf H-J, Hapke U, Bischof G, John U, Meyer C (2019). Diagnostic performance of the alcohol use disorders identification test (AUDIT) in detecting DSM-5 alcohol use disorders in the general population. Drug Alcohol Depend.

[20] Lo-Ciganic W-H, Gellad WF, Gordon AJ, Cochran G, Zemaitis MA, Cathers T (2016). Association between trajectories of buprenorphine treatment and emergency department and in-patient utilization. Addiction.

[21] Busch SH, Fiellin DA, Chawarski MC, Owens PH, Pantalon MV, Hawk K (2017). Cost-effectiveness of emergency department-initiated treatment for opioid dependence. Addiction.

[22] Holzbach R, Stammen G, Kirchhof U, Scherbaum N (2019). The Prescription of anticraving medication and its economic consequences. Eur Addict Res.

[23] Baser O, Chalk M, Rawson R, Gastfriend DR (2011). Alcohol dependence treatments: comprehensive healthcare costs, utilization outcomes, and pharmacotherapy persistence. Am J Manag Care.

[24] Schwarz R, Zelenev A, Bruce RD, Altice FL (2012). Retention on buprenorphine treatment reduces emergency department utilization, but not hospitalization, among treatment-seeking patients with opioid dependence. J Subst Abuse Treat.

[25] Wiercigroch D, Sheikh H, Hulme J (2020). A rapid access to addiction medicine clinic facilitates treatment of substance use disorder and reduces substance use. Subst Abuse Treat Prev Policy.

[26] Corace K, Willows M, Schubert N, Overington L, Mattingly S, Clark E (2020). Alcohol medical intervention clinic: a rapid access addiction medicine model reduces emergency department visits. J Addict Med.

[27] Hu T, Snider-Adler M, Nijmeh L, Pyle A (2019). Buprenorphine/naloxone induction in a Canadian emergency department with rapid access to community-based addictions providers. CJEM.

