# Supplementary material for: Facilitating rapid access to addiction treatment: a randomized controlled trial
Source: Addict Sci Clin Pract. 2021 May 25;16:34. doi: 10.1186/s13722-021-00240-y (PMC8152083; doi:10.1186/s13722-021-00240-y)
Supplement: Supplementary file 1 — Additional file 1: Appendix S1. Eligibility screening questionnaires. [file 13722_2021_240_MOESM1_ESM.docx]

**Additional file 1**

**Appendix S1: Eligibility screening questionnaires**

**Alcohol**

If the patient is not eligible to participate in the H-SOAP study, or decides not to participate, this form will be securely destroyed following this meeting. If the patient is eligible and provides written informed consent to participate in the H-SOAP study, the subject ID # will be written on this form and it will be stored securely at Women’s College Hospital.

Has the patient given verbal agreement to document eligibility screening responses? **Y/N**

Date:
Time:

At this time, is the patient cognitively impaired, due (for example) to intoxication, severe withdrawal or sedation or any other cause? **Y/N**
If yes, please rearrange the interview for a later time or date.

1. In the past 30 days, has alcohol been a problem for you? **Y/N**
2. What problems has alcohol caused for you?
   □ Spending a lot of time drinking
   □ Unable to do some of the things I’m supposed to do
   □ Problems with family, friends or at work
   □ Problems with money, housing or the law
   □ Intoxication
   □ Withdrawal symptoms
3. If yes to number 1, ask the patient to complete the attached AUDIT and score it. **AUDIT score:**
4. (Women aged 18–50) Is there any chance that you are currently pregnant? **Y/N**(*If Yes, ask patient if she will permit you to contact the research coordinator or physician investigator.)
5. The subject is eligible for the study if all criteria below are met:
   □ Responds Yes to 1
   □ Lists at least 1 problem for 2
   □ AUDIT score is 8+
   □ Responds No to 4

**Opioids**

If the patient is not eligible to participate in the H-SOAP study, or decides not to participate, this form will be securely destroyed following this meeting. If the patient is eligible and provides written informed consent to participate in the H-SOAP study, the subject ID # will be written on this form and it will be stored securely at Women’s College Hospital.

Has the patient given verbal agreement to document eligibility screening responses? **Y/N**

Date:
Time:

At this time, is the patient cognitively impaired, due (for example) to intoxication, severe withdrawal or sedation or any other cause? **Y/N**
If yes, please rearrange the interview for a later time or date.

1. In the past 30 days, have opioids been a problem for you? (Opioids include heroin, Percocet, OxyNEO, fentanyl patch, dilaudid, Hydromorph Contin, Tylenol #3, morphine, statex, MS Contin, etc.) **Y/N**
2. What problems have opioids caused for you?
   □ Spending a lot of time using opioids
   □ Strong urge or desire to use opioids
   □ Unable to do some of the things I’m supposed to do
   □ Problems with family, friends or at work
   □ Problems with money, housing or the law
   □ Withdrawal symptoms
3. Are you currently being prescribed methadone or Suboxone (buprenorphine) for treatment of opioid dependence? **Y/N**
4. (Women aged 18–50) Is there any chance that you are currently pregnant? **Y/N**(*If Yes, ask patient if she will permit you to contact the research coordinator or physician investigator.)
5. The subject is eligible for the study if all criteria below are met:
   □ Responds Yes to 1
   □ Lists at least 1 problem for 2
   □ Responds No to 3 and 4
